# Three species of *Hitobia* Kamura, 1992 (Araneae, Gnaphosidae) from south-west China

**DOI:** 10.3897/zookeys.464.8403

**Published:** 2014-12-16

**Authors:** Cheng Wang, Xian-Jin Peng

**Affiliations:** 1 College of Life Sciences, Hunan Normal University, Changsha, Hunan 410081, China

**Keywords:** Ground spider, south-east Asia

## Abstract

Two new species and one new record of the *Hitobia* are described from Gaoligong Mountains, Yunnan Province, China: *Hitobia tengchong* **sp. n.** (male), *Hitobia hirtella* **sp. n.** (male) and *Hitobia makotoi* Kamura, 2011. Distributional data and illustrations of body and copulatory organs are provided. The differences between the new species and their related species are discussed.

## Introduction

The genus *Hitobia* was established by Kamura 1992 with the type species *Micaria unifascigera* Bösenberg & Strand, 1906. A total of 14 species have been reported from south-east Asia only (Platnick 2014). Subsequent papers about this genus were published by scholars from both Chinese and overseas such as Yin et al. (1996), Deeleman-Reinhold (2001), Zhang et al. (2009), Kamura (2011) and so on. Song et al. (2004) and Yin et al. (2012) made detailed studies on Chinese species of *Hitobia* and described 5 new species. Kamura (1992) transferred *unifascigera* from *Poecilochroa* and *asiatica* from *Berlandina* to this genus from Japan. Deeleman-Reinhold (2011) transferred *tenuicincta* from *Ladissa* to this genus from Vietnam. To date, all species of this genus (Platnick 2014) are known in China except for *Hitobia makotoi* Kamura, 2011 occurring in Japan, *Hitobia tenuicincta* (Simon, 1909) from Vietnam and *Hitobia yaginumai* Deeleman-Reinhold, 2001 from Thailand. *Hitobia* is similar to *Litopyllus* Chamberlin, 1922 in the condition of female median spinnerets and male palpal structure, but can be separated from the latter by the slightly recurved posterior eye row, instead of being procurved in *Litopyllus* (Kamura, 1992).

While examining the specimens collected from the Gaoligong Mountains (Yunnan province, south-west China) by the Sino-American Expeditions (1998–2008), one female specimen was identified to be *Hitobia makotoi*, two male specimens were identified to be the members of *Hitobia*, but differ from any other males of the genus. Because of the habits of ground spider and their similar appearance, it is not easy to match male to female in each species, and many species were recorded only with single male or female in a same genus of Gnaphosidae (e. g. *Micaria logunovi* Zhang, Song & Zhu, 2001 based on only one male specimen and *Micaria marusiki* Zhang, Song & Zhu, 2001 based on 2 female specimens). So, we described the two male specimens as two new species. Goal of this paper is to provide the distributional data, illustrations of body and copulatory organs, and the differences between the new species and their related species.

## Material and methods

All specimens were kept in 75% ethanol, examined, measured and drawn with an Olympus SZX16 stereomicroscope and an Olympus BX53 compound microscope. Photos were taken with a digital camera Canon PowerShot G12 mounted on an Olympus SZX16 and compound focus images were generated using Helicon Focus software (3.10 Free).

All measurements were given in millimeters. Leg measurements are giving as: total length (femur, patella + tibia, metatarsus, tarsus). The abbreviations used in text including: AER anterior eye row; ALE anterior lateral eyes; AME anterior median eyes; MOA median ocular area; PER posterior eye row; PLE posterior lateral eyes; PME posterior median eyes. Specimens are deposited in College of Life Sciences, Hunan Normal University.

## Taxonomy

### *Hitobia* Kamura, 1992

#### Hitobia tengchong sp. n.

Taxon classificationAnimaliaAraneaeGnaphosidae

http://zoobank.org/A2EE881F-D6ED-4EA4-8C53-5512D8BC3B00

Figs 1 –8

##### Type material.

**Holotype:** ♂, **China, Yunnan:** Tengchong County, Jietou Township, 8# boundary post of Yakou (25°80.894'N, 98°62.080'E, 2890 m), 23 May 2006, Xingping Wang, Xianjin Peng leg.

##### Etymology.

The specific name refers to the type locality; adjective.

##### Diagnosis.

**Figures 1–4.**
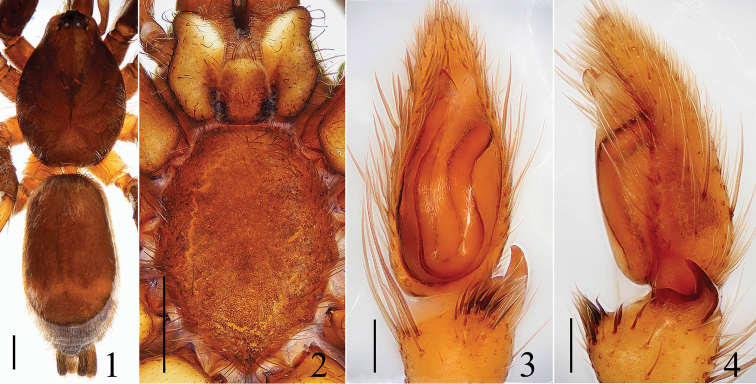
*Hitobia tengchong* sp. n. **1** male body, dorsal view **2** prosoma, ventral view **3** male palp, ventral view **4** male palp, retrolateral view. Scale bars: 0.5 mm (**1**–**2**); 0.1 mm (**3**–**4**).

This new species is somewhat similar to *Hitobia yaginumai* Deeleman-Reinhold, 2001 (see Deeleman-Reinhold 2001: figs 868–874), especially in opisthosoma having a large dorsal scutum, retrolateral tibial apophysis bearing a tuft of long setae on the base, male palp with a obvious conductor, but can be distinguished from the latter by: 1) embolus erect, the tip reached to the position of 11:00 o’clock approximately (Figs 3, 7) versus encircling along the top of bulb prolaterally, the tip reached to the position of 2:00 o’clock in *Hitobia yaginumai*; 2) conductor lamellate in retrolateral view (Figs 4, 8) versus almost semicircular in *Hitobia yaginumai*; 3) retrolateral tibial apophysis hornlike and its apex only extending to the quarter of cymbium in retrolateral view (Figs 4, 8) versus hook-like and its apex extending about to the middle part of cymbium in *Hitobia yaginumai*; 4) abdominal dorsum only with one transverse white stripe (Fig. 1) versus with two additional short longitudinal white stripes on each side except for one transverse white stripe in *Hitobia yaginumai*; 5) chelicerae with 3 promarginal teeth (Fig. 6) versus 2 in *Hitobia yaginumai*.

**Figures 5–8.**
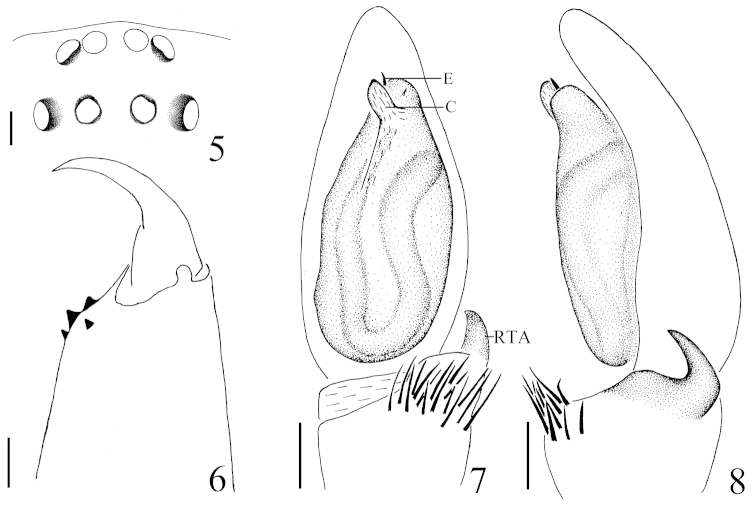
*Hitobia tengchong* sp. n. **5** eye area, dorsal view **6** left chelicera, posterior view **7** male palp, ventral view **8** male palp, retrolateral view. Scale bars: 0.1 mm (**5**–**8**). **C** conductor **E** embolus **RTA** retrolateral tibial apophysis.

##### Description.

**Male:** Total length 5.15. Prosoma 2.29 long, 1.67 wide. Opisthosoma 2.72 long, 1.52 wide. Clypeus 0.05 high. Carapace dark brown, long oval, widest at coxae II and III, covered with some white hair. Cervical grooves, fovea and radial grooves dark brown. AER and PER both slightly recurved, wider posteriorly (Fig. 5). Eyes sizes and interdistances: AME 0.08, ALE 0.08, PME 0.07, PLE 0.09, AME–AME 0.04, AME–ALE 0.01, PME–PME 0.09, PME–PLE 0.09, ALE–PLE 0.14. MOA anterior width 0.18, posterior width 0.22, length 0.25. Chelicerae brown, with 3 promarginal teeth and 1 retromarginal (Fig. 6). Endites yellowish brown, almost parallel (Fig. 2). Labium yellowish brown, longer than wide, ligulate (Fig. 2). Sternum colored as labium, covered with some dark bristles, anterior straight and posterior subacute (Fig. 2). Legs femur, coxae I and II dark brown, others yellowish brown. Trochanters I and II without ventral notch, trochanters III and IV with a shallow ventral notch. Legs spinnation: femur: I, II, III d1-1-0, r0-0-1; IV d0-0-1; patella: I, II, III p0-1-0; IV p0-1-0; tibia: I v1-1-1; II v1-1-1; III d1-0-0, p1-0-0, v1-0-0, r1-1-1; IV d1-0-0, v1-0-1, r0-1-0; metatarsi: I v1-0-0; II v1-0-0, p1-0-0; III d1-0-0, p0-1-0, v1-0-0; IV d1-1-0, p0-1-0, r0-1-0, v1-1-0. Legs length: I 4.65 (1.31, 1.72, 1.02, 0.60), II 4.61 (1.29, 1.70, 1.02, 0.60), III 4.28 (1.02, 1.45, 1.21, 0.60), IV 5.84 (1.71, 2.00, 1.53, 0.60). Dorsum of opisthosoma (Fig. 1) dark brown, long oval, with a large scutum about four-fifths of the whole abdominal length and one transverse white stripe posteriorly, covered with white thin hair. Venter brown.

Male palp (Figs 3–4, 7–8): tibia short, with several long prolatral macrosetae, the retrolateral apophysis hornlike and bearing a tuft of long and curved macrosetae on the base. Bulb elongated, widest at middle part. Embolus thin and short, originating from the prolateral top of bulb, erect, the tip reached to the position of 11:00 o’clock approximately in ventral view. Conductor large relatively, membranous, situated retrolaterally at embolus, lamellate in retrolateral view.

**Female:** Unknown.

##### Distribution.

China (Yunnan).

#### Hitobia hirtella sp. n.

Taxon classificationAnimaliaAraneaeGnaphosidae

http://zoobank.org/67B532D8-9C8E-477A-8339-EFDDE055615C

Figs 9 –16

##### Type material.

**Holotype** ♂, **China, Yunnan:** Nujiang Prefecture, Gongshan County, Pengdang Township, Longpo Village, 12.5 air km of Gongshan (27°85.608'N, 98°68.448'E, 1550 m), 4–7 July 2000, Hengmei Yan leg.

##### Etymology.

The specific name comes from the Latin *hirtella* (with macrosetae), referring to the three thick setae on the cymbial tip.

##### Diagnosis.

**Figures 9–12.**
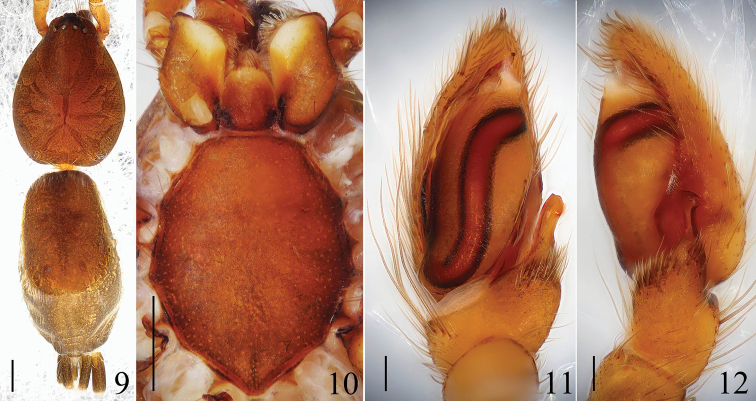
*Hitobia subhirsuta* sp. n. **9** male body, dorsal view **10** prosoma, ventral view **11** male palp, ventral view **12** male palp, retrolateral view. Scale bars: 0.5 mm (**9**–**10**); 0.1 mm (**11**–**12**).

This new species resembles *Hitobia shaohai* Yin & Bao, 2012 (see Yin et al. 2012: figs 631a–h) in having a similar size of dorsal scutum, retrolateral tibial apophysis bearing a cluster of bristles on the base, but can be separated by: 1) conductor visible in ventral view (Figs 11, 15) versus invisible in *Hitobia shaohai*; 2) retrolateral tibial apophysis longer, stronger, the distal end not bifurcated (Figs 11–12, 15–16) versus with two rami in *Hitobia shaohai*; 3) opisthosoma dorsum without obvious markings (Fig. 9) versus with one median pale transverse white stripe in *Hitobia shaohai*; 4) chelicerae with 3 promarginal teeth (Fig. 14) versus with 2 in *Hitobia shaohai*.

**Figures 13–16.**
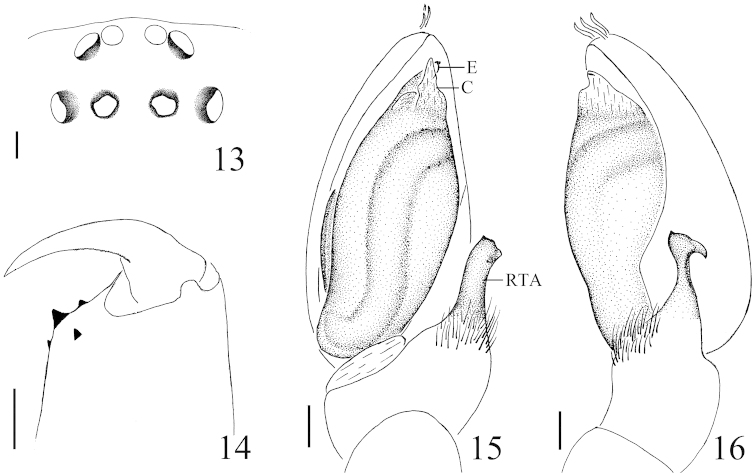
*Hitobia subhirsuta* sp. n. **13** eye area, dorsal view **14** left chelicera, posterior view **15** male palp, ventral view **16** male palp, retrolateral view. Scale bars: 0.1 mm (**13**–**16**). **C** conductor **E** embolus **RTA** retrolateral tibial apophysis.

##### Description.

**Male:** Total length 5.30. Prosoma 2.33 long, 1.75 wide. Opisthosoma 2.85 long, 1.63 wide. Clypeus 0.06 high. Carapace brown, long oval, widest at coxae II and III, covered with some white hair. Fovea, cervical grooves and radial grooves dark brown. AER and PER both slightly recurved, wider posteriorly (Fig. 13). Eyes sizes and interdistances: AME 0.08, ALE 0.10, PME 0.09, PLE 0.09, AME–AME 0.05, ALE–AME 0.01, PME–PME 0.10, PME–PLE 0.10, ALE–PLE 0.14. MOA anterior width 0.21, posterior width 0.25, length 0.29. Chelicerae dark brown, with 3 promarginal teeth and 1 retromargianal tooth (Fig. 14). Endites yellowish brown, almost parallel (Fig. 10). Labium brown, longer than wide, ligulate (Fig. 10). Sternum brown, with some dark bristles, anterior straight and posterior subacute (Fig. 10). Legs femur, coxae I and II dark brown, others yellow. Trochanters I and II without ventral notch, trochanters III and IV with a shallow ventral notch. Leg spination: femur: I, II, III d1-1-1; IV d1-0-0; tibia: I v2-2-1; II v2-2-1; III d1-0-0, p0-1-0, v0-2-0; IVv1-2-1, r1-1-0; metatarsi: Iv0-1-0; II v1-0-0; III d0-1-0, p1-0-1, v2-0-0, r1-0-0; IV d1-0-0, p1-0-1, r0-1-0. Legs length: I 4.85 (1.50, 1.79, 0.91, 0.65), II 4.82 (1.50, 1.76, 0.91, 0.65), III 4.7 (1.32, 1.51, 1.22, 0.65), IV 6.11 (1.75, 2.00, 1.71, 0.65). Dorsum of opisthosoma (Fig. 18) brown, long oval, with three pairs of muscle impressions and a scutum about three-fifths of whole abdominal length, without obvious markings. Venter pale brown.

**Figures 17–20.**
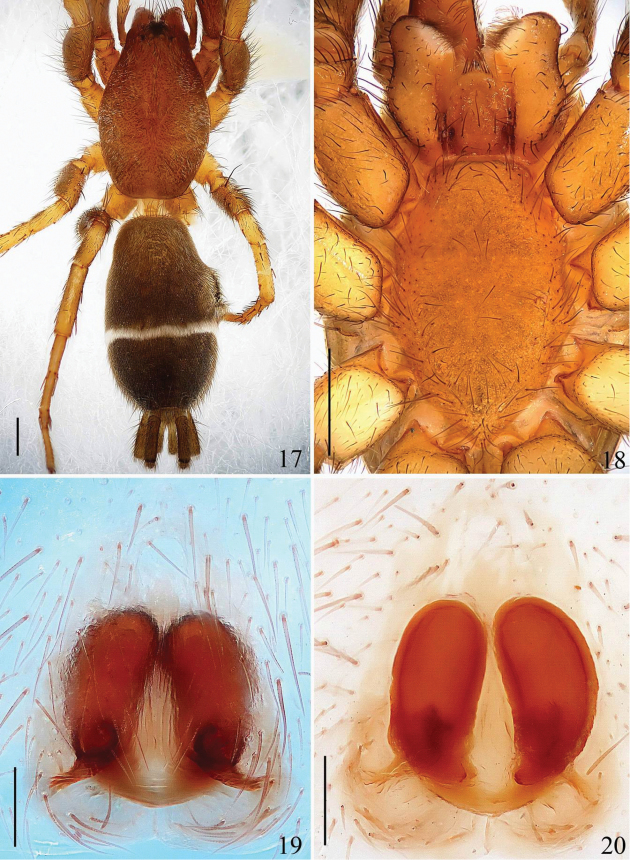
*Hitobia makotoi* Kamura, 2011 **17** female body, dorsal view **18** prosoma, ventral view **19** epigyne, ventral view **20** vulva, dorsal view. Scale bars: 0.5 mm (**17**–**18**); 0.1 mm (**19**–**20**).

Male palp (Figs 11–12, 15–16): tibia short and strong, with several long prolatral macrosetae, the retrolateral apophysis long and bearing a tuft of long bristles on the swollen base. Cymbial tip with three thick setae. Embolus thin, twisted in middle part and the distal part covered by large conductor is, membranous, almost triangular in ventral view.

**Female:** Unknown.

##### Distribution.

China (Yunnan).

#### Hitobia makotoi

Taxon classificationAnimaliaAraneaeGnaphosidae

Kamura, 2011

Figs 17 –23

Hitobia makotoi Kamura, 2011: 104, f. 3–7 (Df).

##### Material examined.

1♀, **China, Yunnan:** Tengchong County, Qingshui Township, Rehai area, Liangyong Village (24°94.919'N, 98°44.921'E, 1450 m), 1 June 2006, D. H. Kavanaugh, R. L. Brett, Dazhi Dong leg.

##### Description.

**Figures 21–23.**
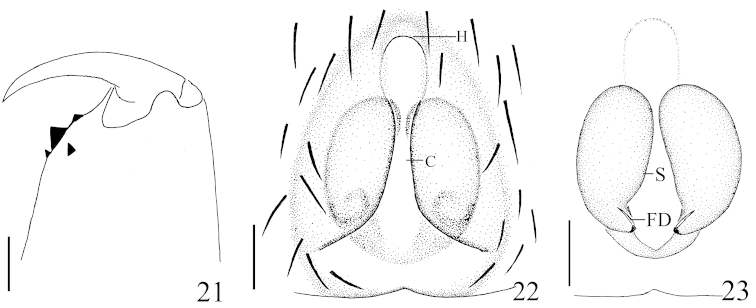
*Hitobia makotoi* Kamura, 2011, **21** left chelicera, posterior view **22** epigynum, ventral view **23** vulva, dorsal view. Scale bars: 0.1 mm (**21**–**23**). **C** concavity **FD** fertilization ducts **H** hood **S** spermathecae.

**Female:** Total length 5.08. Prosoma 2.28 long, 1.45 wide. Opisthosoma 2.63 long, 1.47 wide. Clypeus 0.06 high. Carapace blackish brown, long oval, widest at coxae II and III, covered with some white hair. Fovea, cervical grooves indistinct. AER and PER both slightly recurved, wider posteriorly. Eyes sizes and interdistances: AME 0.09, ALE 0.09, PME 0.07, PLE 0.08, AME–AME 0.03, AME–ALE 0.01, PME–PME 0.08, PME–PLE 0.09, ALE–PLE 0.13. MOA anterior width 0.18, posterior width 0.21, length 0.23. Chelicerae dark brown, with 3 promarginal teeth and 1 retromarginal (Fig. 21). Endites narrowed medianly and slightly convergent apically, almost parallel (Fig. 18). Labium yellowish brown, longer than wide, ligulate (Fig. 18). Sternum colored as labium, with some dark bristles, anterior straight and posterior subacute (Fig. 18). Legs femur, trochanters I and II, coxae I and II brown, others light yellow. Trochanters I and II without ventral notch, trochanters III and IV with a shallow ventral notch. Legs spinnation: femur: I, II v1-1-1; III d1-1-1, p0-0-1; IV d1-1-1, r0-0-1; patella: I, II, III, IV; tibia: I v1-1-1; II v1-0-0; III d1-1-0, p1-0-0, v1-2-1, r1-1-1; IV v1-0-2, r0-1-1; metatarsi: I d0-1-0; II v1-0-0; III d0-1-1, p0-1-1, v1-0-2, r1-0-1; IV d1-1-0, p0-1-0, v0-2-1, r0-0-1. Measurements of legs: I 4.36 (1.35, 1.65, 0.75, 0.61), II 4.28 (1.30, 1.62, 0.75, 0.61), III 4.21 (1.15, 1.31, 1.00, 0.75), IV 5.30 (1.75, 1.85, 1.00, 0.70). Dorsum of opisthosoma (Fig. 17) grayish brown, long oval, with three pairs of muscle impressions at central part and one narrow transverse white stripe posteriorly, covered with recumbent hair. Venter pale brown. Spinneret cylindrical, median spinneret long, with spigots on distal part, blackish brow.

Epigyne (Figs 19–20, 22–23) longer than wide, with a distinct anterior hood, and shallow longitudinal concavity in median part. Spermathecae big, elongated and the distal parts close to each other.

**Male:** Unknown.

##### Distribution.

China (Yunnan), Japan (Amami-öshima Is.).

**Figure 24.**
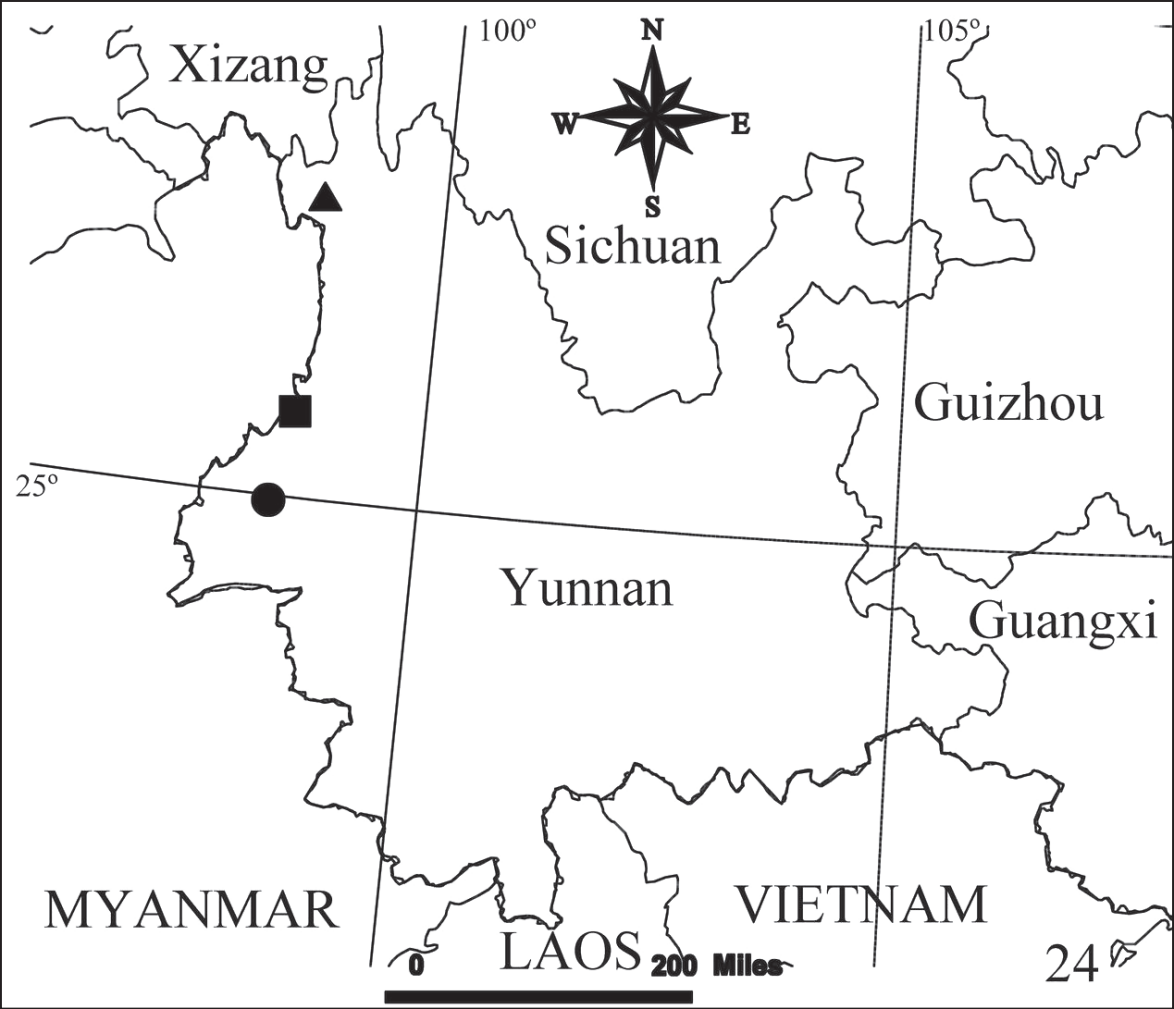
Distribution records of the three species of genus *Hitobia* from south-west China. ▲ *Hitobia hirtella*; ■ *Hitobia tengchong*; ● *Hitobia makotoi*.

##### Comments.

Although the spermathecae of the specimen are smaller, the distal parts close to each other (almost parallel to each other in the original description of Kamura (2011)), the following characters of the specimen are almost as same as those described in the original description: the position and form of stripes on the dorsum of opisthosoma; epigyne with a distinct anterior hood, a shallow longitudinal concavity in median part, copulatory opening indistinct; hence the specimen was identified as *Hitobia makotoi* Kamura, 2011.

## Supplementary Material

XML Treatment for Hitobia tengchong

XML Treatment for Hitobia hirtella

XML Treatment for Hitobia makotoi
